# Interventions to optimize prescribed medicines and reduce their misuse in chronic non-malignant pain: a systematic review

**DOI:** 10.1007/s00228-020-03026-4

**Published:** 2020-10-30

**Authors:** Aziza Alenezi, Asma Yahyouche, Vibhu Paudyal

**Affiliations:** School of Pharmacy, Institute of Clinical Sciences, College of Medical and Dental Sciences, Sir Robert Aitken Institute for Medical Research, University of Birmingham, Edgbaston, B15 2TT Birmingham, England UK

**Keywords:** Chronic non-malignant pain, Chronic pain, Medication optimization, Opioid use

## Abstract

**Purpose:**

Sub-optimal opioid prescribing and use is viewed as a major contributor to the growing opioid crisis. This study aims to systematically review the nature, process and outcomes of interventions to optimize prescribed medicines and reduce their misuse in chronic non-malignant pain (CNMP) with a particular focus on minimizing misuse of opiates.

**Methods:**

A systematic review of literature was undertaken. Search of literature using Medline, EMBASE and CINAHL databases from 2000 onwards was conducted. Screening and selection, data extraction and risk of bias assessments were undertaken by two independent reviewers. Narrative synthesis of the data was conducted.

**Results:**

A total of 21 studies were included in the review, of which three were RCTs. Interventions included clinical (e.g. urine drug testing, opioid treatment contract, pill count), behavioural (e.g. electrical diaries about craving), cognitive behavioural treatment and/or educational interventions for patients and healthcare providers delivered as a single or as a multi-component intervention. Medication optimization outcomes included aspects of misuse, abuse, aberrant drug behaviour, adherence and non-adherence. Although all evaluations showed improvement in medication optimization outcomes, multi-component interventions were more likely to consider and to have shown improvement in clinical outcomes such as pain intensity, quality of life, psychological states and functional improvement compared to single-component interventions.

**Conclusions:**

A well-structured CNMP management programme to promote medicines optimization should include multi-component interventions delivered by a multidisciplinary team of healthcare professionals and target both healthcare professionals and patients. There was heterogeneity in definitions applied and interventions evaluated. There is a need for the development of clear and consistent terminology and measurement criteria to facilitate better comparisons of research evidence.

**Supplementary Information:**

The online version of this article (10.1007/s00228-020-03026-4) contains supplementary material, which is available to authorized users.

## Introduction

Chronic non-malignant pain (CNMP) is defined by the International Association for the Study of Pain as persistent pain regardless of normal tissue healing, for 3 months or more [1]. CNMP is a complex and variable interplay between biological, psychological and social factors. It is categorized by the World Health Organization (WHO) as a chronic disease because, it may last a lifetime, can lead to functional impairment, is irreversible and requires patient rehabilitation, frequent medical care and supervision [1]. CNMP prevalence estimates greatly vary, ranging from 8% to 60% in the general population [2, 3]. Adverse clinical, humanistic and economic consequences for patients, families and society have been reported [4].

Medication optimization is defined ‘as a person-centred approach to safe and effective medication usage and to guarantee that patients get the optimal outcomes from their medications’ [5]. Analgesics including opioids are cornerstone in CNMP treatment. Optimizing medicines use in CNMP includes preventing and mitigating ‘problematic behaviour’ in relation to the use of opioids and gabapentinoids in addition to promoting adherence to the prescribed regimen [5]. ‘Problematic behaviour’ is aberrant behaviour suggestive of addiction, misuse or abuse of prescribed opioids for use or administration other than intended by the prescriber and is commonly reported among CNMP patients [6]. A previous literature review demonstrates that up to 50% of CNMP patients are known to demonstrate problematic behaviours [6].

Healthcare professionals face the problem of effectively treating CNMP while preventing inappropriate medications use and as well as promoting appropriate prescribing and de-prescribing. Therefore, clinicians need strategies and assessment tools to help them weigh the risks and benefits of CNMP treatment. Previous systematic reviews have looked at interventions to prevent or reduce opioids misuse [7, 8]. The concept of medicines optimization as a whole has not been investigated. The aim of this study was to systematically review the nature, process and outcomes of interventions used to optimize prescribed medicines use and reduce their misuse in CNMP patients.

## Methods

This study was conducted based on the Cochrane Guideline and reported as per Preferred Reporting Items for Systematic Review and Meta-analysis (Electronic supplement 1) [4]. Systematic reviews are protocol driven reviews based on priori objectives and plans for literature search, data collection and analyses. Structured approach to identifying, appraising, and synthesizing all relevant studies on a given topic enables researchers to avoid bias in the review process [9]. The protocol was developed as per the PRISMA-P (PRISMA-Protocol) guideline (registered PROSPERO ID = CRD42018111569).

### Literature search

Electronic searches were conducted in Medline, EMBASE and CINAHL databases using medical subject headings (MeSH) and free text keyword, including medication adherence, patient compliance, adherent, adherence, non-compliant, noncompliance, non-adherent, non-adherence, chronic pain, chronic non-malignant pain and chronic non-cancer pain. The search was limited to 2000 onwards.

### Inclusion and exclusion criteria

Studies in the English language using an intervention aimed at optimizing the appropriate usage of prescribed pain medication among adults with CNMP using pain medication for 3 months or more were included. The following concepts were included in medicines optimization: medication adherence, drug misuse, patient compliance to prescribed treatment, including deviation and misuse, adherence/ non-adherence, compliance/non-compliance or safe and effective use of medicines. Studies eligible for inclusion included randomized controlled trials (RCTs), nonrandomized and quasi experimental studies, case-control, cohort and cross-sectional studies published in the English language. Eligible participants were 18+ years with CNMP. Studies involving participants from care homes, residential homes or hospices were excluded as these patients were assumed not to be self-administering their medicines. Both medicines optimization outcomes such as misuse of opioid and clinical outcomes such as pain score, depression and anxiety scale and functional interference were considered.

### Screening and selection

Two independent investigators (AA and VP) screened the study abstracts according to the inclusion and exclusion criteria. Any conflicts arising regarding the selection were resolved by a third investigator (AY), by discussion or by obtaining the study’s full text.

### Data extraction and quality assessment

A data extraction form was developed. Data on study characteristics, intervention types and outcome measures were extracted. The two authors independently extracted the data and any disagreement was resolved through discussion or by a third reviewer. For studies utilizing RCT designs, the risk of bias was assessed using the outline criteria of the Cochrane Handbook for Systematic Reviews of Interventions [9]. The critical appraisal skills programme (CASP) was used for the quality assessment for the rest of the studies.

Two authors (AA and VP) independently performed the assessment, any disagreements were resolved through discussion with a third author (AY). Narrative synthesis of the data was conducted because the studies were heterogeneity in definitions, types of interventions, and measurements and reporting methods such as meta-analysis should be conducted when included studies are sufficiently homogeneous in participants involved, interventions, terminology and outcomes to provide a precise summary [10].

## Results

**Fig. 1:**
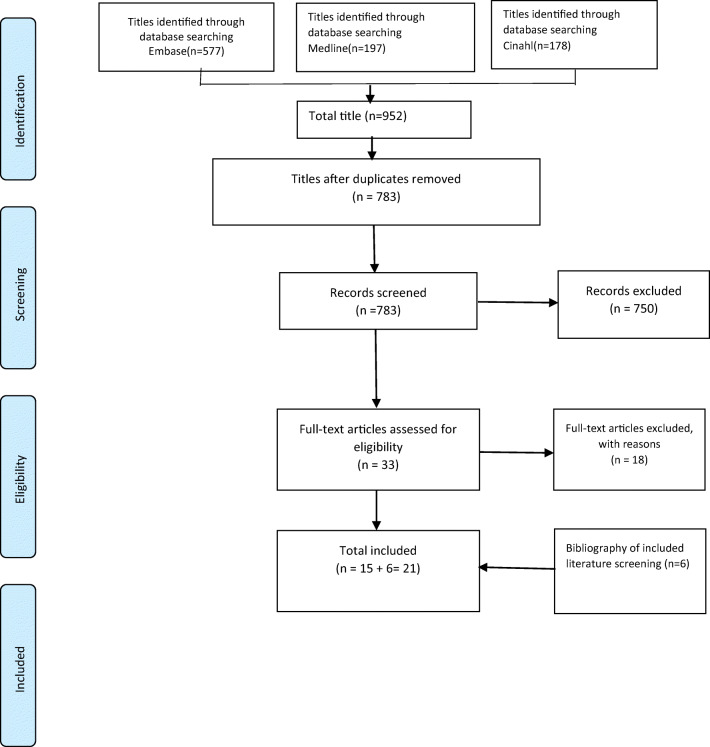
PRISMA diagram of literature search and inclusion process. PRISMA: preferred reporting items for systematic reviews and meta-analysis

From 952 records initially identified, 21 studies met the inclusion criteria (Fig. 1). The reasons for study exclusion are detailed in the Supplementary Material. Of the 21 included studies, three were RCTs and 18 were observational designs.

### Methodological quality

#### RCTs

The three RCTs were assessed for risk of bias using the Cochrane tool [9]. All three studies [11–13] were judged to be at high risk of selection bias, due to the lack of clarity provided regarding the sequence generation process and allocation concealment of participants (Table 1).

**Table 1:** Risk of bias within included randomized controlled trials (RCTs)

QUESTION	Was the allocation sequence adequately generated?	Was allocation adequately concealed?	Was knowledge of the allocated intervention adequately prevented during the study?	Were incomplete outcome data adequately addressed?	Are reports of the study free of suggestion of selective outcome reporting?	Was the study apparently free of other problems that could put it at a high risk of bias?
Jamison, et al. (USA) [11]	Low risk	Unclear risk	Unclear risk	Low risk	Unclear risk	Unclear risk
Timmerman et al. (Netherlands) [12]	Low risk	Unclear risk	Unclear risk	Low risk	Unclear risk	Unclear risk
Wilson et al. (USA) [13]	Unclear risk	Unclear risk	Unclear risk	Low risk	Unclear risk	Unclear risk

#### Other study designs

The quality of the quasi-experimental (*n* = 18) was measured using the CASP quality assessment tool (Table 2). All 18 studies addressed a clear study aim. The majority (*n* = 14) did not provide adequate information on the recruitment procedure (n = 14). Five studies mentioned how the outcomes were measured, four studies did not and the others (*n* = 9) were unclear (Table 2).

**Table 2:** Quality assessment of included observational studies

	Did the study address a clearly focused issue?	Was the cohort recruited in an acceptable way?	Was the exposure accurately measured to minimize bias?	Was the outcome accurately measured to minimize bias?	Have the authors identified all important confounding factors?	Have they taken account of the confounding factors in the design and/or analysis?	Was the follow up of subjects complete enough?	Was the follow up of subjects long enough?
Study ID (country)								
Katz et al. (USA) [21]	Yes	No	Unclear	Unclear	No	No	Unclear	Unclear
Chelminski et al. (USA) [17]	Yes	Unclear	Unclear	Yes	No	Unclear	Unclear	Unclear
Manchikanti et al. (USA) [23]	Yes	No	No	Yes	No	No	Unclear	Unclear
Manchikanti et al. (USA) [24]	Yes	No	Unclear	Yes	No	No	Unclear	Unclear
Hariharan et al. (USA) [18]	Yes	No	Yes	No	No	No	Yes	Yes
Wiedemer, et al. (USA) [29]	Yes	No	No	No	No	No	Yes	Yes
Bethea, et al. (USA) [14]	Yes	Unclear	Unclear	Unclear	No	No	Unclear	No
Brown et al. (USA) [15]	Yes	Unclear	Unclear	Unclear	No	Yes	Unclear	Yes
Wasan et al. (USA) [28]	Yes	Unclear	Unclear	Yes	Yes	Yes	Unclear	Yes
Andersen et al. (Denmark) [30]	Yes	Unclear risk	Yes	Unclear	Yes	Unclear	Yes	Unclear
Kipping et al. (Germany) [31]	Yes	Unclear	No	Unclear	Unclear	No	Unclear	Unclear
Chang et al. (USA) [16]	Yes	Yes	Yes	Yes	Unclear	Unclear	No	No
Jamison et al. (USA) [11]	Yes	Yes	Yes	Unclear	No	Unclear	Yes	Yes
Jacobs et al. (USA) [19]	Yes	Yes	Unclear	No	No	No	Unclear	No
Talusan,2016^.^(USA) [27]	Yes	Unclear	Unclear	Unclear	Unclear	Unclear	Unclear	Yes
Knezevic et al. (USA) [22]	Yes	Yes	Yes	Yes	No	No	Unclear	Unclear
Schell et al. (USA) [26]	Yes	Unclear	Unclear	Unclear	Unclear	Unclear	Unclear	Unclear
McCann et al. (USA) [25]	Yes	Unclear	Unclear	Unclear	Unclear	Unclear	Yes	Yes

#### Characteristics of included studies

The studies were published between 2003 and 2018. Eighteen were conducted in the United States [11, 13–29], and the remainder in Denmark [30], Germany [31] and the Netherlands [12]. Three studies were RCT (Table 3) [11–13]. Nine studies used prospective cohort design [15, 17, 20, 23, 24, 28, 30]. Seven were retrospective cohort studies [18, 19, 21, 22, 25–27] one was a cross-sectional design [31] and one was pre-post interventional design without a control arm [16]. Six studies were conducted in primary care settings [17–19, 25, 27, 29], four in pain centres [23, 24, 28, 31], seven in outpatient’s pain clinics [14, 15, 22, 26, 29, 31], two recruited patients from primary care and pain clinics [16, 20] and one recruited patient from primary care and an internet site (Table 3) [13].

**Table 3:** Characteristics of included studies

Study ID (country)	Aim	Study design & methodology	*Description of study population*			Study duration and setting	Medicines taking behaviour as defined in the study
			Sample size	Participant characteristics	Pain type(s)		
Katz et al. (USA) [21]	To describe the results of urine toxicology screens and behavioural monitoring in patients with chronic non-cancer pain prescribed opioids for daily use.	Retrospective cohort study using medical records to review patients’ drug-taking behaviours, and urine toxicology reports.	**Baseline:**122 **Follow-up:**122	**Age mean:**45 **SD:** 11 **Female %:**49	**Condition(s):** low back pain, neuropathic pain. **Pain type:** nociceptive & neuropathic. **Medication(s):** Opioids, non-opioids.	**Study Duration:** 45 months **Study Setting:** two pain centres.	**Behavioural “issues”– ** lost or stolen prescriptions, consumption in excess of prescribed dosage, visits without appointments, multiple drug intolerances and allergies, and frequent telephone calls).
Chelminski et al. (USA) [17]	To examine the effects of multi-disciplinary pain management on the outcomes of pain, disability, depression and substance misuse.	Prospective cohort study using medical records and urine samples of the recruited patient.	**Baseline:** 85 **Follow-up**:63	**Age mean**:50 **SD:**NA **Female %**:40	**Condition(s):** Back pain, mixed aetiologies, chronic headache. **Pain type**: nociceptive &neuropathic. **Medication(s):** Opioids.	**Study Duration:** 3 months. **Study Setting:** primary care.	**Substance misuse–** detection of cocaine or amphetamines; Procurement of opioids from more than one provider; Diversion of opioids; UTS negative on at least two occasions for prescribed opioids; UTS positive on at least two occasions for opioids not prescribed by our practice.
Manchikanti et al. (USA) [23]	To evaluate the effect of implementing of a controlled substance agreement, including periodic review of medications, urine testing, pill counts and verification of medication information on controlled substance.	Prospective cohort study with historical controls using medical records and urine samples of the recruited patient.	**Baseline:** 566 **Follow-up:**500	**Age mean:**48.5 **SD:** NA **Female %:**59	**Condition(s):** chronic pain. **Pain type:** Nociceptive. **Medication(s):** Opioids.	**Study Duration:** Not clear **Study Setting:** Multidisciplinary pain management centre.	**Abuse–**patient receiving controlled substances from any other place or source other than the prescribing physician at our centre, with the exception of controlled substances for acute injuries unrelated to the problem being treated, or for emergencies.
Manchikanti et al. (USA) [24]	To study the prevalence of illicit drug use in patients receiving opioids with adherence monitoring combined with random urine drug testing.	Prospective cohort study with historical controls using medical record and urine samples of patients.’	**Baseline:** 566 **Follow-up**:500	**Age mean**:48.5 **SD:** NA **Female %:**59	**Condition(s):** chronic pain. **Pain type:** Nociceptive. **Medication(s):** Opioids.	**Study Duration:** Not clear **Study Setting**: Multidisciplinary pain centre	**Illicit drug use** – use of an illicit drug during the month prior to the survey interview.
Hariharan et al. (USA) [18]	To describe the long-term outcomes of a medication contract agreement for patients receiving opioid medications in a primary care setting.	Retrospective cohort study using medical records.	**Baseline:** 332 **Follow-up:**330	**Age mean**:49 **SD:**NA **Female %:**48	**Condition(s):** Back pain, Fibromyalgia Joint Diseases. **Pain type**: Nociceptive. **Medication(s):** Opioids.	**Study Duration:** 5 years **Study Setting**: Primary care	**Compliance–** patients receiving stable doses of contracted medications at prescribed intervals and adhering to the conditions of the contract agreement.
Wiedemer, et al. (USA) [29]	To measure the impact of a structured opioid renewal programme for chronic pain run by a nurse practitioner and clinical pharmacist.	Naturalistic prospective outcome study using medical records and questionnaire.	**Baseline: 335Follow-up:** 171	**Age mean:** NA **SD:** NA **Female %:** NA	**Condition(s):** chronic pain. **Pain type:** not specified. **Medication(s):** Opioids.	**Study Duration:** 24-month. **Study Setting:** a primary care clinic.	None.
Bethea, et al. (USA) [14]	To investigate alliance development for patients targeting chronic pain and opioid analgesic abuse. The study hypothesizes growth in alliance would be lower for patients with greater substance abuse severity.	A prospective cohort study using Questionnaires.	**Baseline:** 36 **Follow-up:**25	**Age mean:**41.40 **SD:**9.81 **Female %:**44	**Condition(s):** chronic pain. **Pain type:** not specified. **Medication(s):** Opioids.	**Study Duration:** 12 weeks **Study Setting:** Veterans Administration-based pain clinic.	**Abuse** or **“aberrant medication-taking behaviours”** (AMTBs)– include not taking pain medication as prescribed, reporting lost/stolen prescriptions, and using supplemental opioids obtained from unauthorized or illegal sources.
Jamison, et al. (USA) [11]	To see if close monitoring and cognitive behavioural substance misuse counselling could increase overall compliance with opioids.	A randomized trial using questionnaires and structured interview.	**Baseline:** 66 **Follow-up**:62	**Age mean:**47.7 **SD:**7.14 **Female %:**43.5	**Condition(s):** back pain. **Pain type:** Nociceptive. **Medication(s):** Opioids.	**Study Duration:** 6 months **Study Setting:** Veterans Administration-based pain clinic	**Substance misuse**– the use of any drug in a manner other than how it is indicated or prescribed. **Substance abuse**: is defined as the use of any substance when such use is unlawful, or detrimental to the user or others.
Brown et al. (USA) [15]	To evaluate potential for aberrant- drug related behaviours among patients with chronic, moderate- to-severe pain and to determine investigator compliance with universal precautions approach to pain management.	Prospective open-label, nonrandomized, non-comparative, multi-centre observational study using questionnaires	**Baseline:**1487 **Follow-up**:561	**Age mean**:53 **SD:** Non **Female %:**57	**Condition(s):** Arthritis, skeletal muscle disease. **Pain type:** Nociceptive. **Medication(s):** Opioids.	**Study Duration:** 12 weeks **study Setting:** pain clinic in a hospital.	N**on-adherence- **Sedation, frequent request of early refill, increase dose without authorization, repeated lost or stolen prescription or unexpected positive or negative urine drug screen.
Wasan et al. (USA) [28]	To examine the relationships between self-reports of craving, and opioid misuse and to characterize self-reports of craving in patients prescribed opioids for chronic pain.	A prospective, longitudinal, descriptive, cohort study using Questionnaires.	**Baseline:** 66 **Follow-up**:62	**Age mean:**47 **SD:** NA **Female %:**43.5	**Condition(s):** Back or neck pain. **Pain type:** Nociceptive. **Medication(s):** Opioids.	**Study Duration:** 6 months **Study Setting:** pain clinic in a hospital.	**Substance misuse**- the use of any drug in a manner other than how it is indicated or prescribed. Substance abuse is defined as the use of any substance when such use is unlawful, or when such use is detrimental to the user or others.
Andersen et al. (Denmark) [30]	To investigate the association between attachment insecurity, pain, disability, distress and the use of opioids.	A prospective study using questionnaires and patients’ journals.	**Baseline:** 72 **Follow-up:**72	**Age mean:** 43 **SD:** 7.1 **Female %:**86.	**Condition(s):** Lower back and legs Upper back and arms **Pain type:** Nociceptive **Medication(s):** opioids	**Study Duration:** 13-week **Study Setting:** pain clinic in a hospital.	None
Kipping et al. (Germany) [31]	To investigated patients’ reliability regarding their report of any current pain medication use.	A cross-sectional study using urine sample from recruited patients.	**Baseline:**343 **Follow-up**:343	**Age mean**:55.2 **SD:** 14.8 **Female %:**45%	**Condition(s):** low back, neuropathic, or viscera **Pain type:** nociceptive/ neuropathic **Medication(s):** opioids, non-opioids, co-analgesics.	**Study Duration:** at the first visit **Study Setting:** pain clinic.	**Compliance-** patients’ drug intake behaviour in accordance with prescribed instructions.
Chang et al. (USA) [16]	To test the effect of office-based motivational interviewing (MI) on prescription opioid adherence in older adults with chronic pain.	Pre- and post-design using interviews and questionnaires.	**Baseline:** 33 **Follow-up:**30	**Age mean**:59.1 **SD**: 6.1 **Female %:**50	**Condition(s):** Chronic pain. **Pain type**: Not specified. **Medication(s):** Opioids.	**Study Duration:** 4-week **Study Setting**: primary care office and a pain management clinic.	**Non-adherent–** if they do not take medication at the proper dosage indicated or more than prescribed.
Wilson et al. (USA) [13]	To measure the effects of an Internet-based self-management program among patients who receive opioids from a primary care provider.	A randomized controlled trial.	**Baseline: 114 Follow-up:92**	**Age mean:51** **SD: NA** **Female %:78**	**Condition(s):** back pain, fibromyalgia, arthritis, **Pain type:** Nociceptive, neuropathic **Medication(s): Opioids**	**Study Duration:** 4-week. **study Setting:** primary care and a pain management clinic.	None
Jamison et al. (USA) [20]	To investigate the benefits of interventions designed to track potential opioid misuse and to improve practitioner confidence in managing CNMP patients.	A Prospective Longitudinal Controlled Trial using questionnaires.	**Baseline:** 253 **Follow-up:**253	**Age mean**:53.7 **SD:**10.6 **Female**: %:59.7	**Condition(s):** Low back pain. **Pain type:** Nociceptive. **Medication(s):** Opioids.	**Study Duration:** 6 months. **study Setting:** primary care and Specialist clinics.	None
Jacobs et al. (USA) [19]	To assess the effect of two clinical pharmacists using a telephone risk assessment clinic to promote safe opioid prescribing.	A retrospective study using pharmacy and medical records.	**Baseline:** 608 **Follow-up:** 148	**Age mean**:64 **SD**: NA **Female** %:	**Condition(s):** Chronic pain **Pain type:** Not specified **Medication(s):** Opioids	**Study Duration:** 3 months. **study Setting:** Primary Care Clinic.	**Aberrant behaviour–** taking an opioid medication in any manner other than as prescribed.
Talusan,et al^.^(USA) [27]	To develop, implement, and evaluate an Opioid Monitoring Clinic as a clinical referral system within the primary care	A retrospective chart review of participants.	**Baseline: 61** **Follow-up: 39**	**Age mean: 53** **SD: NA** **Female %:7**	**Condition(s):** Back pain Pain type: Nociceptive**.** **Medication(s):** Opioids.	**Study Duration:** 6 months. **study Setting:** Primary Care Clinic.	**None.**
Timmerman et al. (Netherlands) [12]	To investigate the effect of medication-specific education on pain medication adherence.	A randomized controlled trial.	**Baseline:**112 **Follow-up:**96	**Age mean**:52.5 **SD:**15.1 **Female** %:68.4	**Condition(s):** Chronic pain **Pain type:** Nociceptive, neuropathic **Medication(s):**): opioids, non-opioids, co-analgesics.	**Study Duration:** 12 weeks **study Setting:** Centre for Pain Medicine.	**Underuse-** missing a dose once a week up to every day, **overuse** was defined as admitting to taking more medication than prescribed**. Non-adherence** was defined as presence of underuse and/or overuse of prescribed medication.
Knezevic et al. (USA) [22]	To assess the compliance of chronic opioid consuming patients in an outpatient setting and evaluate if utilizing repeated urine drug testing could improve compliance.	Retrospective analysis of prospectively collected data using urine samples and patient medical charts.	**Baseline:**500 **Follow-up:**500	**Age mean**:53.93 **SD:**14.76 **Female** %:54	**Condition(s):** Neck, back, Shoulder and knee pain,, peripheral neuropathy **Pain type:** nociceptive /neuropathic **Medication(s):** opioids.	**Study Duration:** not clear **study Setting:** Outpatient pain management clinic.	**Compliance**– urine sample was only positive for opioid medications that were prescribed by our pain clinic without the identified presence of any illicit drug.
Schell et al. (USA) [26]	To evaluate the effect of interventions on the appropriate monitoring of chronic opioid therapy as well as the appropriate management of high-risk chronic opioid therapy >200 mg MEDD.	A retrospective data analysis of clinical impact of these interventions.	**Baseline:222Follow-up: 58**	**Age mean:NA** **SD:NA** **Female %:NA**	**Condition(s): Chronic pain** **Pain type:** Not specified **Medication(s):** Opioids and Benzodiazepine.	**Study Duration: 7 months study Setting: 6** community-based outpatient clinics**.**	**None**
McCann et al. (USA) [25]	To study the impact of a systematic approach that adhered to evidence-based guidelinesfor managing patients with non-malignant cancer pain.	A retrospective study using medical records.	**Baseline:**32 **Follow-up:**29	**Age mean:**66.86 **SD:** NON **Female %**:31	**Condition(s):** Neck, back, Shoulder pain, neuropathy **Pain type**: nociceptive &neuropathic **Medication(s):** opioids.	**Study Duration:** 18 months. **study Setting**: rural primary care practice.	**None**

NA: Not available

#### Medicines optimization goals

Most of the included studies aimed to reduce the use or misuse/abuse of drugs or aberrant drug behaviour or to enhance appropriate use of prescribed opioids in CNMP patients. Four of the included studies aimed to reduce the use or reliance on opioids [13, 19, 25, 30], nine aimed to identify and reduce misuse, abuse of drugs or aberrant drug behaviour [11, 14, 17, 20, 21, 23, 24, 28, 29]. Two studies aimed to improve adherence (*n* = 2) [12, 16] and four focused on patients’ compliance with prescribed opioids [11,18, 22, 31]. Considering pain type and locations, eight studies included both nociceptive and neuropathic pain patients [12, 13, 17, 18, 21, 22, 25, 31]. Eight studies focused on populations suffering from nociceptive pain, including low back pain, arthritis and other musculoskeletal disorders [11, 15, 20, 23, 24, 27, 28, 30]. Five studies did not specify the type of pain or provided insufficient patients’ information [14, 16, 19, 26, 29]. None of the studies focused on other types of analgesics, especially co-analgesic medication (such as antidepressants or anticonvulsants) (Table 3).

#### Medication taking behaviour

The scope of medication taking behaviour was defined differently among the included studies. These included misuse [11, 17, 28], abuse [23], aberrant drug-taking behaviour [14, 19, 21], non-adherence(*n* = 3) [12, 15, 16], compliance (n = 3) [18, 22, 31] and illicit drug use (*n* = 1) [24]. Seven studies did not define any medication taking behaviour [13, 20, 25–27, 29, 30].

Tables 3 and 4 to be included here.

## Type of interventions

### Single component interventions

#### Opioid treatment contract (OTC) with or without urine drug screening (UDS)

A total of 11 studies used single component interventions (Table 4). An OTC is a bilateral contract between the patient and physician that defines each party’s responsibilities. These included OTC alone or combined with urine drug screening to measure patients’ adherence to the contract terms (Table 4). Only three studies published a copy or details of the OTC [15, 17, 18].

**Table 4:** Nature of intervention used by the included studies

Interventions types and components			Single-component intervention (with or without urine drug screening)											Multi-components intervention									
			Katz, et al.[21]	Manh ikanti et al.[24]	Hariran, et al.[18]	Bethea, et al.[14]	Kipping,et al.[31]	Wasn, et al.[28]	Andersen, et al. [30]	Chang, et al.[16]	Wilson, et al.[13]	Timer man, et al. [12]	Knezevic, et al. [22]	Chelin ski, et al.[17]	Manh ikanti, et al.[23]	Wied emer, et al.[29]	Jamis on, et al.[11]	Brown, et al.[15]	Jam ison, et al.[20]	Jacobs, et al.^[19^^]^	Talusan, et al.[27]	Schell, et al.[26]	McCann, et al. [25]
Structured CNMP programme		Urine drug screening (UDS)	**×**	**×**	**×**		**×**						**×**	**×**	**×**	**×**	**×**	**×**	**×**	**×**	**×**	**×**	**×**
		Opioids treatment agreement or contract (OTA/ OTC)			**×**									**×**	**×**	**×**	**×**	**×**	**×**	**×**	**×**	**×**	**×**
	Follow up	Monthly clinical/ physical /pain assessment												**×**			**×**	**×**	**×**	**×**		**×**	**×**
		Risk assessment/	**×**																	**×**	**×**	**×**	
		Prescription monitoring program														**×**				**×**	**×**	**×**	**×**
		Opioid dose adjustments												**×**		**×**				**×**	**×**	**×**	**×**
		Prescribing small quantity/ pill count													**×**	**×**		**×**					
		Healthcare provider-led intervention												**×**		**×**				**×**	**×**	**×**	**×**
	Educational	Patient education										**×**					**×**					**×**	
		Provider education/informatics tool																**×**	**×**			**×**	
	Behavioural	Cognitive, emotional,				**×**		**×**	**×**	**×**							**×**						
		Psychiatric consultation												**×**		**×**	**×**					**×**	
	Technological	Electronic diaries						**×**									**×**						
		Internet based self-management programme									**×**												

OTA = opioid treatment agreements; OTC: opioid treatment contract; UDS = urine drug screen

#### Behavioural interventions

Five studies used different behavioural approaches alone either to reduce misuse, abuse or to reduce patient reliance on opioids, while one aimed to improve patient opioid adherence (Table 4). These include working alliance, which refers to the mutual relationship that grows between the therapist and patient during the treatment period [14], cognitive and behavioural treatments based on attachment orientations, which are defined as moderately stable schemas that regulate emotions and our responses to health behaviour and stressors [30]; in-clinic electronic diaries to answer 25 questions to monitor patients’ craving progress [28]; brief office-based introductory motivational interviews on the adherence to opioid medication [16]; and internet-based pain management programme for targeting behavioural, cognitive, emotional and social factors [13].

#### Educational interventions

Only one study evaluated the effect of specific medication education on patients’ adherence to their medication using a brief video and written instruction about the medication name, dosage, and frequency [12].

### Multi-component interventions

Multi-component interventions were used by ten studies (Table 4). Each intervention included at least three components along with UDS and OTC. Most of these programmes followed the relevant local or national clinical practice guidelines for managing opioid therapy for chronic pain. The components of complex interventions included the following:

1. *Urine drug screening and opioid treatment contract within multi-component intervention*

All the included studies categorized as multi-component interventions incorporated OTC. The studies generally included UDS alongside OTC (Table 4).

2. *Monthly clinical/pain assessment*

Seven studies of complex intervention studies involved a monthly physical or pain assessment (Table 4).

3. *Risk assessment*

Risk assessment was used to help determine risk potential for ‘Aberrant Drug-Related Behaviour’ (ADRB) (Table 3). Three studies used the Screener and Opioid Assessment for Pain Patients-Revised (SOAPP-R) questionnaire, a standardized score tool that can be answered by patients or their providers [19, 26, 27] (Table 4). One study used the opioids/benzodiazepines combination risk assessment tool created by the study team as YES/NO questions [26]. Other studies stated that ADRB was identified by self-reporting or evaluated by the healthcare providers using other tools but did not mention using a standardized questionnaire [19].

4. *Prescription monitoring programme (PMP)*

PMP are state-based electronic databases that are used to track prescriptions, mainly for controlled substances, including benzodiazepines, opioids and amphetamines. Five studies used PMP to optimize pain medication use and reduce inappropriate use (Table 4).

5. *Opioid dose adjustments*

Six studies used average daily Morphine Equivalent Daily Dose (MEDD) adjustment as an intervention to optimize medication use (Table 4).

6. *Prescribing/dispensing small quantities/pill count*

Three studies included pill counts or small quantities of opioids [15, 23, 29]. Pill count was described as done in a random selection method; however, it was not clear who performed the count [23] (Table 4).

7. *Team-based approach*

Six studies used a team-based approach led by one or multiple providers [17, 19, 25–27, 29]. These included the combined skills of clinical pharmacist, internist and psychiatric [17], use of a structured package containing documents to be filled by patients regarding pain history medication use, psychiatric evaluation and educational information [25], a primary care provider run outpatient clinic and aided by dedicated nursing, pharmacy and mental health care providers [26], pharmacists run opioid use clinic and informal consultative services in primary care including the use of telephone [19], multidisciplinary pain management team who met bi-weekly to review cases and recommend treatment plans [29].

8. *Patient education within a multi-component intervention*

Only two studies involved patient education as a component of their interventions [11, 26]. These included printed educational material [26] and group sessions [11].

9. *Provider education within a multi-component intervention*

Three studies educated or trained providers before participating in the studies [15, 20, 26]. These included pain management education and monitoring techniques.

10. *Behavioural interventions within a multi-component intervention*

Only one study offered behavioural intervention as a part of a complex intervention where patients participated in a structured motivational and cognitive behavioural training programme to prevent substance misuse [11].

11. *Psychiatric consultation*

Four studies included a psychiatric consultation plan or a psychiatrist as part of the treatment programme [11, 17, 26, 29]. The studies differed in role and goal of including this component which included psychiatric consultation as support for patients at high risk of misuse, abuse and addiction [11, 17, 29] assessment of patient risk, stability and the presence of contraindications of using an opioid/BZD combination to treat CNMP [26].

12. *Electronic diaries*

Only one study used electronic diaries to monitor cravings among patients and the effect is reported briefly in the study [11].

### Study outcomes

There was substantial heterogeneity on the nature of the outcomes (Table 5). These were systematically categorized as follows:

**Table 5:** Description of intervention

Study ID(country)	Description of intervention	Primary outcome	Primary outcomes-key findings (pre and post interventions)	Secondary outcome	Secondary outcomes–key findings	Author conclusions
**Single-component interventions**						
Katz et al. (USA) [21]	Patients monitored for 3 yrs. with urine drug screen (UDS) and any behaviour suggestive of inappropriate medication use were documented.	Detection of “problem” (either r positive urine toxicology (UDT) or one or more aberrant drug-taking behaviours).	**Medicine optimization outcome:** Of patients with no behavioural issues, Positive UDS alone was 21%, behavioural issues alone were 14%. patients both positive UDS and behavioural issues was 8% of the patients. No aberrant drug behaviours were 57% of patients.	–	–	Requiring a report of behavioural issues and urine toxicology screens creates a more comprehensive monitoring system than either alone.
Manchikanti et al. (USA) [24]	Random urine drug testing for chronic pain patients on chronic opioid therapy	The prevalence of illicit drug use in patients receiving opioids for chronic pain management	**Medicine optimization outcome:** Illicit drug use was evident in 16% of patients including marijuana in 11%, cocaine in 5%, and amphetamines in 2% and decreases in obtaining opioid from multiple providers or sources from 17.8% to 9.2% (ARR, 8.6% [CI, 4.4% to 12.8%]).	–	–	The study showed significant reductions in overall illicit drug use with adherence monitoring combined with random urine drug testing. This study also showed absence of illicit drug use in the elderly.
Hariharan et al. (USA) [18]	Opioid Contract: specified the conditions under which opioids would or would not be prescribed and patient responsibilities which included random urine drug testing.	Describe outcome of a medication contract agreement.	**Medicine optimization outcome:** Contracts discontinued in 37% of patients. Contract cancelled for substance abuse in 17% of patients. 20% discontinued contract voluntarily 38% UDS positive for illicit substances	–	–	A more structured drug testing strategy is needed to identify nonadherent patients.
Bethea, et al. (USA) [14]	An 8-session cognitive behavioural intervention. Sessions 1 and 2 focused on emotional bonds, sessions 3 and 4 focused on patient difficulties with pain control, managing opioid. Session 5 focused on treatment goals and tasks. In sessions 6 and 7, a therapist provided ongoing feedback on treatment progress. In session 8, the therapist and physician met with the patient to review the treatment and inform the patient of the teams’ decision of methadone tapering.	Relationship of the working alliance to treatment success (opioid adherence, and urine toxicology and decreasing pain, functional interference, and substance abuse	**Medicine optimization outcome:** Correlations of the working alliance to opioid adherence failed to reveal statistically significant relationships until the end of treatment (r = .51). *p* = < .05 **Clinical outcome:** Correlations of the working alliance with treatment” outcomes failed to reveal statistically significant relationships between either patient or therapist alliance scores and average pain (r = −.53). *p* = < .05 Functional interference mood, and relationships with others (r = −.43)	–	–	Patient alliance was unrelated to opioid adherence and unauthorized drug use; in contrast, lower therapist alliance was related to poorer adherence and higher levels of unauthorized drug use. Examining “during treatment” outcomes including repeated assessments of pain, functional interference, opioid adherence and urine screening. Correlations failed to reveal statistically significant relationships between either patient or therapist alliance scores and pain ratings until the end of treatment.
Wasan et al. (USA) [28]	Electronic diaries for ratings opioid craving at monthly clinic visits and daily during a 14-day take-home period. The device contained questions from the Brief Pain Inventory (severity, activity, function and mood), medication questions, and location of pain).	The relationship between craving and medication compliance, treatment outcome	**Medicine optimization outcome:** Craving Index (CI mean values): at base line high risk control HRC =26.7, high risk experimental HCE =11.0, at the end of study mean CI values (HRC = 24.5, HRE = 9.6 the differences were statistically significant. *p* = <.05.	–	–	Craving is a potentially important psychological construct in pain patients prescribed opioids, regardless of their level of risk to misuse opioids. Targeting craving may be an important intervention to decrease misuse and improve compliance.
Andersen et al. (Denmark) [30]	Cognitive behaviour therapy that included education in pain theory and management, meditation, relaxation training, visualization, activity-rest scheduling, problem solving, change of maladaptive thoughts, assertive communication, value-based exposure, acceptance strategies supported by handouts.	Attachment anxiety association with pain intensity, association between attachment avoidance and the use of opioids	**Medicine optimization outcome** Use of opioids (pre = 40.41, post = 31.89) *p* = 0.033 **Clinical outcome:** **Pain intensity** (pre = 5.67, post = 5.45) *p* = 0.820 **Depression** (pre = 10.08, post = 9.0) *p* = 0.000 **Physical disability** (pre = 52.64, post = 50.4) *p* = 0.394	–	*–*	Attachment insecurity plays an important role in the chronic pain management. Insecure groups did not differ significantly with respect to pain intensity and physical disability. The insecurely attached group was significantly more anxious and depressed at both pre- and post-treatment. Moreover, the insecurely attached group used significantly more morphine (mg/day) at pre- and post-treatment.
Kipping et al. (Germany) [31]	Blood and urine samples collected and analysed immediately after asking two chronic pain patients’ groups (pain group, surgical group) about other pain medications that they used beside their prescribed pain medication.	The rate of noncompliance, under-reported and over-reported among pain clinic (PG) and presurgical control (SG).	**Medicine optimization outcome** The noncompliance in PG (43.3%) and in SG (24%). *p* = < 0.05 Under-reporting (31% PG; 23% SG) *p* = < 0.05 Over-reporting (11% PG. 2% SG). *p* = < 0.05, frequently under-reported non-opioid analgesics.	–	–	Two types of noncompliance were defined: under-reporting and overreporting. Under-reporting of non-opioid analgesics is the main type of noncompliance.
Chang et al. (USA) [16]	Motivational interviewing face-to-face session focused on increasing participants’ awareness about their use of prescription opioids, followed by weekly phone-delivered follow-up sessions.	The effect of Motivational Interviewing on Prescription Opioid Adherence	**Medicine optimization outcome:** Risk for opioid abuse (pre = 16.40, post 12.60) *p* = < 0.01 Self-efficacy on medication use (pre = 31.19, post = 34. 690. *p* = 0 .009 **Clinical outcome score means:** Depression (pre = 20.24, post = 14.59) *p* = .006 Pain severity worst pain (pre = 8.62, post = 8.62). *p* = 0.862	Treatment satisfaction	Participants reported a good level of satisfaction (mean = 10.1)	Motivational interviewing can be effectively delivered in outpatient settings for older adults who are at risk for opioid misuse. Clinicians could incorporate MI techniques to enhance prescription opioid adherence.
Wilson et al. (USA) [13]	Internet-based self-management programme targeting cognitive, emotional, behavioural and social pain determinants. The programme generates an individualized custom plan based on the result of chronic pain assessment.	Improvements in pain intensity, pain interference and opioid misuse behaviours using The Current Opioid Misuse Measure (COMM).	**Medicine optimization outcome:** Of treatment group COMM score pre = 12.4, post = 9.1)**.** *p* = 0.48 Decreased or stopped opioid =20.9% *p* = 0.04 **Clinical outcome score means:** Pain Intensity (pre = 5.6, post 5.3) *p* = 0.22 Pain Interference (pre = 5.5, post = 5.3). *p* = 0.39 Depression (pre = 12.5, post = 10.3.) *p* = .49	Health Care Utilization	Treatment group participants reported 2.8 total visits to health care providers during the 8-week study period compare to 2.2 in control group. *p* = 0.36	Patients on opioids were able to engage and demonstrate positive outcomes using an Internet-based self-management programme.
Timmerman et al. (Netherlands) [12]	A 5-min educational video contained standardized information about the medication name, frequently used dosing schedules and written medication-specific information.	Medication adherence, education in pain.	**Medicine optimization outcome** The non-adherence rates were 43% at 4 weeks and 49% at 10 weeks in the intervention group. *p* = 0.38 **Clinical outcome score means: In the intervention group** Pain intensity changed from 6.3 to 5.4 Perceived improvement changed from 3.6 to 3.7. *p* = NS	Patient satisfaction	Patient satisfaction changed from 4 to 4.3 in the intervention group. *p* = 0.136	Medication-specific education did increase knowledge of the prescribed therapy but did not improve adherence or treatment outcome parameters.
Knezevic et al. (USA) [22]	Patients were asked to provide supervised urine toxicology specimens during their regular clinic visits, and were asked to do so without prior notification	The compliance with chronic opioid	**Medicine optimization outcome** Repeated UDSs showed that patients’ compliance of patients on opioid medications (63.6%) had improved. *p* = 0.383	*–*	*–*	Repeated UDS can improve compliance of patients on opioid medications and can improve overall pain management.
**Multi-component interventions**						
Chelminski et al. (USA) [17]	Structured clinical programme that combined the skills of internists, clinical pharmacists, and a psychiatrist, monthly follow-up by a nurse, pain contracts, medication titration and psychiatric consultation.	Reducing substance misuse and improve pain, depression, function scores.	**Medicine optimization outcome:** Substance misuse was identified in 32%. **Clinical outcomes Average pain score:** (pre = 6.5, post = 5.5. *p* = 0.003 **Depression score** changed from 24 to18. *p* = 0.003 **Disability index** changed from 47 to 39. *p* = < 0.001	–	**–**	Substance misuse and depression were common, and many patients left the programme when they were no longer prescribed opioids. Effective care of patients should include rigorous assessment and treatment of these comorbid disorders and intensive efforts to ensure follow up.
Manchikanti et al. (USA) [23]	Pain patients on chronic opioid therapy signed controlled substance agreements. Adherence monitoring was carried out by appropriate history, periodic evaluation of appropriate intake of drugs, random drug testing, and pill counts	Reduction in opioid abuse	**Medicine optimization outcome** Opioid abuse reduced by 50% post intervention = NA	–	–	Adherence monitoring, including controlled substance agreements and various periodic measures of compliance, was associated with a 50% reduction in opioid abuse.
Wiedemer,et al. (USA) [29]	A structured opioid renewal programme for chronic pain run by a nurse practitioner (NP) and clinical pharmacist.	Adherence to opioid treatment agreements (OTA) and use of UDS	**Medicine optimization outcome:** The number of OTAs increased by 70% The number of UDS increased by 93%.45% patients adhered to the OTA and resolved their aberrant behaviours, (38%) self-discharged, (13%) were referred for addiction treatment, and (4%) with consistently negative UDS were weaned from opioids.	Reduction in ER and unscheduled PCP visit and cost.	ER visits reduced by 72.7% *p* = NA unscheduled PCP visits reduced by 59.6% Pharmacy cost reduced from $129,793 to $5236. Provider Satisfaction was 84%.	An NP/clinical pharmacist-run clinic, supported by a multi-specialty team, can successfully support a primary care practice in managing opioids in complex chronic pain patients, both in changing abnormal illness behaviour (walk-ins, medication complaints) and in freeing up more time to deal with important medical problems.
Jamison, et al. (USA) [11]	Patients at high-risk of misusing opioids were under close monitoring by implementing the terms of a controlled substance agreement and were asked to participate in a structured cognitive behavioural training program for prevention of substance misuse.	Increase opioids compliance using the Drug Misuse Index (DMI) and urine drug screening.	**Medicine optimization outcome** **Positive (DMI) %** among subjects, High-Risk Control (HRC) = 73.7, High-Risk Experimental (HRE) 26.3. *p* = <0.05 **Urine Screens:** Normal (%) in (HRC)64.7, (HRE) 88.2. *p* = <0.05 **Clinical outcomes:** **Average pain** (HRC =65.32, HRE = 57). *p* = <0.001 **Depression** (HRC = 9.06, HRE = 6.06.) *p* = <0.01 **Activity interference** (HRC = 67, HRE = 63.73. *p* = <0.05	Patients satisfaction with treatment	The high-risk experimental group rated the compliance interventions as generally helpful. The subjects rated the individual counselling sessions on 10 scale (mean = 8.61) and compliance checklists (mean = 8.13)	The results of this study demonstrate support for the benefits of a brief behavioural intervention in the management of opioid compliance among chronic back pain patient at high-risk for prescription opioid misuse.
Brown et al. (USA) [15]	Morphine sulphate extended release capsules, a treatment agreement, card for obtaining/tracking prescriptions, screener and opioid assessment for patients with pain revised, pill count, pain-patients follow-up tool, urine drug testing.	Evaluate and monitor aberrant behaviour Using The screener and opioid assessment for patient with pain Revised (SOAPP-R) UDS.	**Medicine optimization outcome:** At the baseline 47%were considered low risk for opioid misuse/abuse, 52% moderate, and 1% high risk. 90% of the patients remine at the same level of risk at the end of the study. **positive Urine drug screen**% changed from 14 to 10%. P=NA	Investigator compliance with the use of universal precaution (UP) to pain management.	A total of 64% of investigators were compliant with UP, and there was tendency to assign patient to lower level than the protocol specified.	Most patients at primary care were categorized as at least moderate risk of opioid misuse/abuse at baseline. The plan for patients at moderate risk included counselling, monitoring for aberrant drug related behaviour. Patients at high risk for aberrant drug related behaviours were withdrawn from study.
Jamison et al. (USA) [20]	A structured opioid therapy protocol of monthly monitoring and compliance checklists. Patients were contacted by telephone once a month and were asked to complete the assessments.	Opioid compliance	**Medicine optimization outcome:** **Medication compliance** (pre = 51.5, post = 60.8%). **Opioid use for pain** (pre = 87.0%, post = 79%) **Clinical outcome score means:** **Pain scores** (pre = 7.0, post = 6.7). **Activity interference (**Daily routine pre = 6.862, post = 6.7).	Practitioner confidence in identifying patients at risk for misuse and Patients satisfaction	**Patients satisfaction** was (74.2%). **Confidence in opioid prescribing** (pre = 53.5%, post = 36.2%) *P* < 0.01 **Confidence in identifying patients at risk for misuse of pain medication** (pre = 42.9%, post = 63.9%) *P* < 0.05	The patients reported greater compliance with their opioid medication. Many PCPs still lacked confidence in managing pain patients and reported reluctance to prescribe opioids for chronic noncancer pain. This study demonstrates the benefits of careful monitoring of chronic pain patients and need for pain management support within primary care.
Jacobs et al. (USA) [19]	Clinical pain pharmacists reviewed patients’ chart to assess recent opioid use, side effects, laboratories values, and aberrant behaviours and then contacted patients by phone two weeks prior to the prescription renewal date. Based on an assessment, pharmacists documented recommendations on the appropriateness of a patient’s opioid regimen, adjunctive nonopioid medications, side effect management, the frequency of UDS, provider follow-up and laboratory monitoring.	Pharmacist-led telephone risk assessment clinic effect on opioid using in primary care clinic	**Medicine optimization outcome:** Recommendations to change opioid regimens = (32.4%). Decrease the MEDD of opioids (33.3%). Discontinuation of opioid therapy (15; 22.7%), **OTC** use (pre = 4.7%, post = 64.8%) *p* < .0001 **UDS** (pre = 62.8%, post 79.7%) *p* = 0 .002 PDMP report (pre = 30.4%, post = 100%) *p* = < .0001	–	–	A clinical pharmacist-run telephone risk assessment promotes safe use of chronic opioid therapy through improved monitoring and identification of aberrant behaviours in more than one third of assessed patients.
Talusan, et al. (USA) [27]	An advanced practice registered nurse led opioid monitoring clinic (OMC) which included protocol for urine drug screening, PMP database access, reports of opioid dispensing from Pharmacy Care line, clinic referral template allowing communication between primary care providers and OMC.	Reduction in the Morphine Equivalent Dose (MED) and identified abuse, the use of illicit drugs.	**Medicine optimization outcome** Opioids discontinued in 36%. *p* value = NA Reduction of (MED)59%, *p* = <0.001. The mean MED/d (pre-54 mg/d, 22 mg/d)	Level of satisfaction among primary care providers (PCP)	**PCP satisfaction** = 100% with the Monitoring Clinic service. **PCP following the guidelines for opioid** = 90%. **PCPPDMP access** = 54%. **UDS ordered** = .93% **And** significant pharmacy cost savings.	Opioid Monitoring Clinic can be an effective programme to help identify abuse and misuse of prescription opioids among high-risk patients and can improve patient safety and provider satisfaction.
Schell et al. (USA) [26]	Multidisciplinary pain oversight committee to facilitate appropriate management of chronic opioid therapy (COT) using urine drug testing a prescription drug monitoring programme (PDMP), patient education and provider education.	Appropriate opioid use (reduction in opioid dose)	**Medicine optimization outcome** Opioid dose per patient decreased 20% from baseline. Patients who received an opioid and benzodiazepines decreased 41.7% during the study period.	–	. -	Multidisciplinary pain oversight committee led to increase appropriate COT monitoring and appropriate management of high-risk patients.
McCann et al. (USA) [25]	Structured management of opioid medication started by listing CNMMP patients on opioids from electronic health record of a single rural practitioners then notifying them 3 months before the change in practice. The notification letter explained options for the patients were to continue opioid, manage their pain without opioids or be referred to another provider.	Patients weaning from opioids and reduction in opioid dose	**Medicine optimization outcome** **Wean from opioids** = 38% **Continued opioid =** 53%. Transferred care = 9%. **Mean morphine equivalent** mg/day decrease from (30.61 mg/day) to (17.01 mg/day) (*p* = .0397; CI, 0.68 to 26.51) **Clinical outcome score means:** **BPI Pain Scale (0 to 10) (mean)** = (pre 5.75 post 6.20) **BPI Quality of Life Scale** (0 to 10) (pre 5.84 post 6.11) 95% CI (1.44 to 0.55)	–	–	Intervention provided a high degree of compliance with controlled substance regulations and is associated with a reduced number of opioid prescriptions. Patients on lower doses of opioid medication are more likely to wean their use with this model.

Abbreviations: *COMM* current opioid misuse measure, *HRC* high risk control, *HRE* high risk experimental, *MED* morphine equivalent dose, *OTA* opioid treatment agreements, *PCP* primary care providers, *p* significance level, *PG* pain group, *r* correlation values, *SG* presurgical group, *UDS* urine drug screen, *NA* not available, NS = nonsignificant

Medicine optimization

- Appropriate use of pain medication: Twelve studies aimed to enhance appropriate use, three measured patients’ adherence [12, 14, 16], seven studies measured the reduction of patient’s opioids dose or reliance on opioids [13, 19, 25–27, 29, 30], four measured patients’ compliance [20, 22, 28, 31].
- Inappropriate use of pain medication: Twenty studies aimed to reduce the problematic medication-taking behaviour including misuse, abuse, illicit drug use, seven of these measured aberrant drug behaviours [11, 15, 18, 19, 21, 24, 29], seven measured abuse [11, 14, 16, 18, 23, 27, 29] and six measured misuse [11, 13, 16, 17, 20, 27].
- Self-discharge from chronic opioid treatment

Some patients voluntarily discontinued chronic opioid therapy after implementing a structured opioid programme observed in three studies [18, 25, 27].

#### Clinical outcomes

Only nine studies assessed clinical outcomes such as pain intensity or functional improvement, depression and anxiety [11–14, 16, 17, 20, 25, 30]. One study measured patients’ quality of life using the quality of life scale [25] (Table 5).

#### Other outcomes utilized

These included patient satisfactions [11, 12, 16, 20], reduction in utilization of health care services [13, 29], healthcare costs [27, 29], provider confidence in managing CNMP patients, compliance with universal precautions [20], adherence to clinical guidelines [15, 19, 20, 26, 27, 29, 30] and provider satisfaction [27, 29].

#### Impact of the interventions

UDS was used in simple intervention and complex interventions as the only objective tool to detect inappropriate drug-taking behaviours. The use of UDS was associated with significant reduction in inappropriate medication use [11, 17, 22, 24, 31] (Table 5). For example, in a study which aimed to identify controlled substance abuse in patients receiving prescription opioids showed a statistically significant decreases in obtaining opioids from multiple providers or sources from 17.8% to 9.2% (ARR, 8.6% [CI, 4.4% to 12.8%]) [23]. One study concluded that UDS should not be used alone and should be used with a comprehensive monitoring system [21].

Three studies that were conducted in a pain speciality setting and used UDS and OTC found these were associated with improvements in detecting inappropriate drug-taking behaviours but did not measure clinical outcomes such as pain intensity and depression score [21, 23, 24]. Two studies that used the same sample of patients and used historical control participants concluded that UDT and OTC significantly reduced the problematic behaviour by detecting patients who were obtaining opioids from multiple providers [23, 24]. Behavioural interventions showed that these interventions are beneficial in reducing the inappropriate use of medication, pain intensity and depression score and in improving patient compliance [14, 16, 30]. Another study suggested that careful monitoring of patients’ medication cravings can reduce the inappropriate use of medication and the level of pain experienced is weakly associated with craving opioids [28]. Only one study used special medication educational intervention alone and found a non-significant effect on medication optimization and no significant effect on pain intensity, depression and functionality interference [12].

Overall, treatment programmes incorporating multidisciplinary teams were associated with decreased risk of opioid misuse and improved functioning and pain condition [11, 14, 17, 25, 28]. One study that used a pharmacist and nurse-led interdisciplinary approach in advising primary healthcare providers, prescription management and patient monitoring using UDS in the opioid renewal clinic showed that approximately half who were referred for complexity, including history of substance abuse or need for opioid rotation or titration, continued to adhere to the opioid treatment plan [29]. While in another study that aimed to support providers and change prescribing behaviours and improve their adherence to clinical guidelines, reported that opiate use was successfully discontinued in nearly one in ten patients [19]. Generally the structured programme were associated with a reduction in the inappropriate use of medication but clinical outcomes such as depression, pain, and quality-of-life scores were stable during study time were rarely reported [25] (Table 5). A study which included daily patient visits to a primary care physician clinic and involved verification of the controlled substance contract, UDS, board of pharmacy monitoring, pain-targeted history and physical, calculation of the average morphine equivalents used showed that 8% elected to wean opioids, 53% continued opioid medication and 9% transferred care. Mean morphine equivalent mg/day was the prime determinant for ability to wean (17.01 mg/day) compared with maintaining (30.61 mg/day) (*p* = .0397; CI, 0.68 to 26.51) [25]. Patients maintaining opioid treatment showed no statistically significant change in clinical outcomes such as quality of life and depression scale point compared with the baseline [25]. Another study conducted in a multidisciplinary primary care programme monitored patients with psychiatric comorbidity showed an improvement in patients’ mood and pain scores but, a high dropout rate reported among patients with misuse behaviour was observed [17]. An RCT consisting of monthly electronic diaries, urine toxicology screens and medication adherence counselling for six months reported an improvement to prescribed regimen among high risk patients adherence and patients satisfaction (Table 5) [11].

## Discussion

This study aimed to systematically review the nature and outcomes of interventions to optimize the use of prescribed medication among CNMP patients. An array of different types of single component and multi-component complex interventions were being evaluated to optimize medication use. A variety of definitions were used for medication optimization with a range of other outcomes evaluated which included clinical outcomes, costs, patients and healthcare satisfaction and quality of life.

The results demonstrate an inadequate number of high-quality studies focusing on medication optimization among these patients. The majority of studies (*n* = 10) had a limited sample size and were often conducted in a single setting. Studies differed in recruitment methods and participant inclusion criteria. In addition, there was substantial heterogeneity in the types of populations being studied, their co-morbidities and medications. Studies were of low to moderate quality. It was often unclear which component targets which behavioural issue. A lack of consistency in defining the appropriate use of pain medication was identified.

Most of the studies categorized as simple interventions aimed either to detect or reduce the problematic taking behaviours without taking into consideration their effects on patients’ clinical outcomes such as pain intensity, depression, anxiety and relapse after stopping their medication [18, 21–23, 31]. A previous qualitative study suggested that healthcare providers commonly believed that OTAs were useful for provider self-protection, but they do not prevent opioid misuse [32].

The multi-component interventions included in this review were often based on the United States’ own chronic pain management guidelines such as Department of Defence Clinical Practice Guideline for Management of Opioid Therapy for Chronic Pain [26, 27]. Such multi-component interventions were shown to be more comprehensive and targeted detection and reduction of problematic medication-taking behaviour, taking into consideration patients’ physical and psychological states, as well as their satisfaction [26, 27]. However, the complexity of these interventions makes it unclear to conclude which components targeted which behaviour. Such understanding is needed in order to personalize the interventions for the CNMP population which may differ in various way and from one demographic region to another. For example, patients with a history of addictions and other substance use disorder may need different monitoring than those without such issues [11, 15, 17]. Safe and effective pain management requires knowledge of the principles of chronic opioid therapy as well as effective assessment of risks associated with opioid abuse, addiction and diversion using multi-component interventions. An earlier systematic review specifically focused on opioid misuse also addressed that multi-component interventions that targeted both patients and healthcare professionals are essential to minimize their misuse for CNMP patients [33].

The lack of literature regarding interventions for non-opioid medication and the heterogeneity of studies are major limitations of the published literature.

### Implications for practice

The review suggests that the most promising approach to increase the appropriate use of prescribed pain medication among CNMP patients is a structured opioid clinic with multidisciplinary approach. Clinician ability to appropriately prescribe opioids to CNMP patients with or without problematic medication taking behaviour can be achieved with an appropriate level of understanding and using the components of the complex interventions including UDS and OTC and personalizing pain management according to patient need. Random drug testing might be more suitable to detect misuse and abuse than regular testing and save more money. Well-structured chronic pain program/structured opioid clinic designed to support and improve healthcare providers, communication skills and knowledge for all healthcare providers should be implemented. More attention should be paid to the costs and benefits and of interventions and the impact on clinical staff on the burden as this is potentially important to inform policy and practice decisions [27].

Collaborative relationships and communication between different healthcare settings are important to facilitate medication optimization. Providing high quality, patient-centred care and ensuring patient safety for CNMP patients who are on chronic opioid therapy requires collaboration among healthcare providers [34]. Clinicians need to attend more to the negative impact of psychiatric and behavioural issues on the use of opioids. Additionally, they should initiate discussions with patients who are being prescribed opioids in order to evaluate their medication-taking behaviours and treatment effectiveness, as well as to screen for their risk of prescription opioid misuse and provide intervention as needed. Clinicians must be aware CNMP patients face multiple physical, psychological and social challenges that may impede them from taking their medications as prescribed [35–37].

### Strengths and limitations

This is the first systematic review to include a wide variety of studies on the nature and outcomes of interventions used to optimize medicines use in CNMP patients. The search was limited to three databases and the English language. The search criteria did not include specific chronic pain type or diagnoses, which might have led to the omission of studies.

### Implications for research

There is a need for further studies on interventions to optimize the use of prescribed pain medication, applying high methodological standards and including randomized controlled designs. There is also a need for future studies to better define misuse and other problematic medication-taking behaviour and related complications of opioid therapy, to develop diagnostic procedures for these disorders. Moreover, behavioural issues should be categorized according to their seriousness and their effect on the patient’s quality of life. The studies should also report changes in outcome measurements such as pain levels and psychosocial, physical and occupational functioning and patient’s satisfaction at different time points, rather that only before and after the intervention implementation. The validity and reliability of measurement tools should be carefully tested and explicitly reported. Both interventions and tests should be tailored to specific CNMP populations such as patients with different types of pain, comorbid psychiatric conditions and different behavioural issue. Future efforts should be directed at RCT to examine structured pain management programs that would determine whether reported effects are real examined durability and promote their sustainability. There is also a need for the development of clear and consistent terminology and measurement criteria to facilitate comparisons of research evidence. Research should assess the drawbacks of applying these interventions. Future interventions should include collaborative and structured opioid clinic approaches using nurse, pharmacist or a primary care physician who must be trained in delivering a motivational interview or educational interventions.

## Conclusion

A well-structured CNMP management programme to promote medicines optimization should include multi-component interventions delivered by a multidisciplinary team of healthcare professionals and target both healthcare professionals and patients. There was heterogeneity in definitions applied and interventions evaluated. There is a need for the development of clear and consistent terminology and measurement criteria to facilitate better comparisons of research evidence.

## Supplementary Information

ESM 1 (DOCX 21 kb)
